# Investigating whether serum IGF-1 and IGFBP-3 levels reflect the height outcome in prepubertal children upon rhGH therapy: LG growth study database

**DOI:** 10.1371/journal.pone.0259287

**Published:** 2021-11-01

**Authors:** Minsun Kim, Eun Young Kim, Eun Young Kim, Cheol Hwan So, Chan Jong Kim

**Affiliations:** 1 Research Institute of Clinical Medicine of Jeonbuk National University-Biomedical Research Institute of Jeonbuk National University Hospital, Jeonju, Korea; 2 Department of Pediatrics, Jeonbuk National University Medical School, Jeonju, Korea; 3 Department of Pediatrics, Chosun University, College of Medicine, Gwangju, Korea; 4 Department of Pediatrics, Kwangju Christian Hospital, Gwangju, Korea; 5 Department of Pediatrics, Wonkwang University School of Medicine, Wonkwang University Hospital, Iksan, Korea; 6 Department of Pediatrics, Chonnam National University Hospital, Chonnam National University Medical School, Gwangju, Korea; Sackler School of Medicine, Tel Aviv University, ISRAEL

## Abstract

Serum insulin-like growth factor-1 (IGF-I) and IGF binding protein-3 (IGFBP-3) levels can be used to monitor the safety of recombinant human growth hormone (rhGH) therapy. In this study, we evaluated the changes in serum IGF-I and IGFBP-3 levels during rhGH therapy as a marker of height outcome in prepubertal children. Totally, 705 prepubertal children with short stature were enrolled from the LG Growth Study Database. Data for three groups of subjects were obtained as follows: Idiopathic GH deficiency (IGHD; n = 486); idiopathic short stature (n = 66); small for gestational age (n = 153). Serum IGF-I and IGFBP-3 levels at the baseline and after the 1^st^ and 2^nd^ year of rhGH therapy, as well as the Δheight standard deviation score (SDS), were obtained. Δheight SDS after the 1^st^ and 2^nd^ year of rhGH therapy had notably increased compared to that at the baseline for all three groups. IGF-I and IGFBP-3 levels in all three groups were significantly increased compared to those at the baseline (p <0.001). Δheight SDS was positively correlated with ΔIGF-1 SDS after the 1^st^ year of therapy, **Δ**IGFBP-3 SDS after the 2^nd^ year of therapy in the IGHD group, and ΔIGF-I SDS and ΔIGFBP-3 SDS after the 2^nd^ year of therapy (p < 0.05), regardless of whether the height at the baseline was a covariate. The increase in IGF-I and IGFBP-3 levels during rhGH therapy was related to the growth response in children with IGHD. Therefore, it may be valuable to measure the change in serum IGF-I and IGFBP-3 levels, especially the latter, during rhGH treatment to predict the growth response upon long-term treatment.

## Introduction

Recombinant human growth hormone (rhGH) therapy is widely used for short stature due to various causes, such as chromosomal/genetic abnormalities, small for gestational age (SGA), idiopathic short stature (ISS), and idiopathic GH deficiency (IGHD) [1]. Depending on the disease, the response of increased height has been reported, and many researchers are striving to find a suitable factor that can reflect the safety and efficiency of rhGH [1–5]. The most reliable factors that reflect growth hormone levels is the insulin-like growth factor (IGF)-I [4, 6].

Serum IGF-I and IGF binding protein (IGFBP)-3 levels depend on GH levels under normal physiological conditions [7]. IGF-I concentration is the most widely used parameter for monitoring and adjusting rhGH therapy [8]. With the current measurement method, it is difficult to measure free IGF-I, which is the active form, due to several technical difficulties [3]. Therefore, we need to focus on IGFBP-3, which is the main IGFBP that binds to 90% or more of circulating IGF-I, forming a large ternary complex with acid-labile subunits and IGFs [3, 9]. Although IGFBP-3 is a carrier protein of IGF-I, regulating the activity of IGF-I, the relationship is vague in rhGH therapy [6]. Despite IGFBP-3 normally circulating in a partially fragmented form because of a specific proteolytic enzyme such as pregnancy-associated plasma protein A2 (PAPP-A2) [10], the commonly used ELISA method for its detection cannot distinguish between the intact and cleaved forms [11]. As the fragmented IGFBP-3 cannot easily bind with IGF-I, the measurement of IGFBP-3 level does not reflect the real binding capacity for IGF-I. Therefore, we analyzed GH-IGF-I-IGFBP-3 levels except for IGF-I/IGFBP-3 molar ratios. Although there are a few studies about IGF-I and IGFBP-3 being useful monitoring indexes for determining the efficiency of rhGH, their results have been ambiguous. Furthermore, only a limited amount of previous clinical data has been reported comparing the growth response to long-term rhGH therapy using IGF-I and IGFBP-3 levels in ISS and SGA cases to those in IGHD [12–14].

In this study, we aimed to investigate the changes in serum IGF-I and IGFBP-3 levels during rhGH therapy in prepubertal children with IGHD, ISS, and SGA and their efficiency in predicting the growth response during the first 2 years of rhGH therapy.

## Materials and methods

### Patients

**Fig 1:**
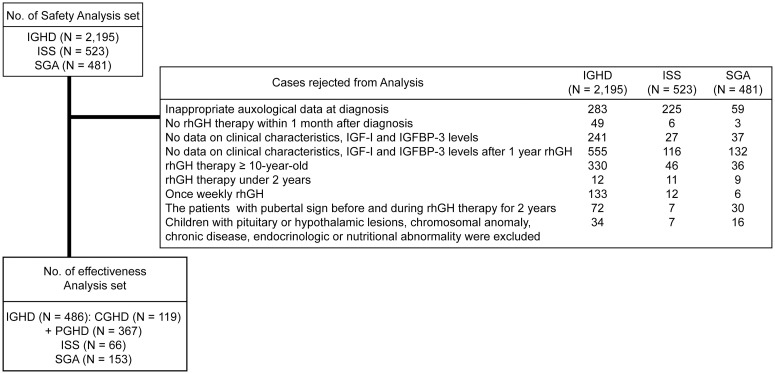
Flow chart of subjects included in this study. GH, growth hormone; rhGH, recombinant human GH; CGHD, complete GH deficiency; IGHD, idiopathic GH deficiency; ISS, idiopathic short stature; SGA, small for gestational age; IGF-I, insulin like growth factor I; IGFBP-3, IGF binding protein 3; PGHD, partial GH deficiency.

This study included data from the LG Growth Study (LGS) [15]. LGS is a multi-center, open-label, and non-interventional study. Clinical and laboratory records of 3199 prepubertal children with IGHD (n = 2195), ISS (n = 523), and SGA (n = 481), who had received rhGH therapy (Eutropin^®^ inj., and Eutropin^®^ Pen inj., LG Chem Ltd., Korea) for ≥ 2 years between February 2001 and February 2020, were initially enrolled. The inclusion criteria were as follows: 1) Height below the third percentile according to the 2017 Korean National Growth Charts [16] at the time of hospital visit for IGHD and ISS cases, and at birth and at the time of hospital visit for SGA cases; 2) Two separate GH stimulation test results using insulin, clonidine, L-arginine, L-dopa, or glucagon; 3) children with a peak GH level less than 5 ng/mL (complete IGHD, CGHD) and between 5 to 10 ng/mL (partial IGHD, PGHD) [17–21] were classified as growth hormone deficient and those with that more than 10 ng/mL as ISS and SGA. The exclusion criteria were as follows: 1) Inappropriate auxological data at diagnosis; 2) no rhGH therapy within 1 month after diagnosis; 3) no clinical data, for serum IGF-I and IGFBP-3 levels before and 1 year after rhGH therapy; 4) age at the beginning of the therapy ≥ 10 years; 5) rhGH therapy length under 2 years; 6) weekly rhGH (Eutropin^®^Plus inj., LG Chem Ltd., Korea); 7) patients with a pubertal sign before or during the 2-year rhGH therapy; 8) children with pituitary or hypothalamic lesions, chromosomal and genetic anomalies, and chronic diseases, as well as endocrinological, skeletal or nutritional abnormalities. The total number of patients included in this study in three different groups were 468, 66, and 153 for IGHD, ISS, and SGA groups, respectively (Fig 1). A total of 705 children with prepubertal short stature that had received rhGH therapy over 24 months were enrolled from the LG Growth Study Database and separated into three groups as follows: IGHD (n = 486), ISS (n = 66), and SGA (n = 153). IGHD group included 119 CGHD patients and 367 PGHD patients.

### Auxological and clinical data

Anthropometric measures and medical histories, including sex, chronological age (CA), bone age (BA), height, weight, body mass index (BMI) (kg/m^2^), mid-parental height (MPH), pubertal state, rhGH dose (mg/kg body weight/week), peak GH serum levels in GH stimulation tests, and serum IGF-I and IGFBP-3 levels, were obtained from the LGS database. The data were collected from their respective data management systems, where all participating centers provided laboratory data generated following standard procedures [22]. The standard deviation score (SDS) values of height, weight, and BMI were converted using the 2017 Korean National Growth Charts for children and adolescents [16]. MPH (cm) was calculated as follows: For girls, (paternal height − 13 + maternal height) / 2; for boys, (paternal height + maternal height + 13) / 2. Then MPH was converted to SDS values. BA was assessed using radiography images of the left hand using the Greulich-Pyle method [14] by the treating physician. The pubertal stage was determined according to Marshall and Tanner [23]. Testicular volume was measured using the Prader orchidometer. Serum IGF-I and IGFBP-3 concentrations (ng/mL) were obtained by multiple researchers. SDS values were calculated according to age- and sex-specific references of Korean children [24]. The difference (Δ) of clinical and laboratory data before and after the treatment was calculated as the change between the two values. The growth response (Δheight, ΔIGF-I and ΔIGFBP-3 SDS) was measured 12 ± 3 and 24 ± 3 months after the treatment. We considered a Δheight SDS of < 0.4 and < 0.3 after the 1^st^ year of therapy as a poor response for children with IGHD, and ISS/SGA, respectively [3, 16, 17].

### Ethics approval and consent to participate

The protocol was approved by the Institutional Review Board of Jeonbuk National University Research Council (IRB no: 2011-10-018). Written informed consent was obtained from all patients and their parents/legal guardians before enrolment at each institute as part of the LGS.

### Statistical analysis

All statistical analyses were performed using SPSS (ver. 23.0, IBM Corp., Armonk, NY, USA). The values before and after 1 and 2 years of treatment were compared using analysis of variance (ANOVA) for repeated measures followed by a posteriori Tukey test or Wilcoxon’s rank test. Among baseline, 1-year and 2-year rhGH therapy, Δheight SDS, as well as serum ΔIGF-I and ΔIGFBP-3, were analyzed using paired t-test. One-way ANOVA or Kruskal-Wallis test was performed to compare the clinical and laboratory findings for the three groups. Comparisons between poor and good growth response groups were assessed using an independent t-test or Wilcoxon’s rank-sum test, depending on the data distribution. Pearson correlation analysis was used to evaluate the association among Δheight SDS and serum ΔIGF-I and ΔIGFBP-3. Analysis of covariance (ANCOVA) between Δheight SDS during 2^nd^ year of rhGH therapy and ΔIGF-I-ΔIGFBP-3 as fixed factor and height at baseline as a covariate was performed. Statistical significance was set at *p* < 0.05. Results are presented as mean ± standard deviation.

## Results

### Comparison of baseline clinical characteristics and laboratory findings

The clinical and laboratory features of prepubertal children with IGHD, ISS, and SGA before treatment and are shown in Table 1. IGHD group comprised CGHD (n = 119) and PGHD (n = 367) sub-groups. Serum IGF-I and IGFBP-3 levels and peak GH level with the GH stimulation test were significantly decreased in CGHD group compared with PGHD (*p* < 0.05), whereas the sex ratio, age, BA, height, weight, BMI, initial rhGH dose did not differ among the groups. According to the results of comparison among IGHD, ISS and SGA; sex ratio, age, height and IGFBP-3 level were not different (p > 0.05). BA, IGF-I SDS, initial rhGH dose, and peak GH level in the GH stimulation test were significantly low, and weight and BMI were showed the opposite trend in the IGHD group (*p* < 0.05) compared with ISS and SGA groups.

**Table 1:** Clinical features of children with IGHD, ISS, and SGA at the beginning of GH treatment.

		IGHD				ISS (n = 66)	SGA (n = 153)	p-value (IGHD vs ISS vs SGA)
		CGHD (n = 119)	PGHD (n = 367)	*p*-value (CGHD vs PGHD)	Total (n = 486)			
Sex, n (%)	Male	76 (63.87)	220 (59.95)	0.4463	296 (60.91)	41 (62.12)	84 (54.90)	0.3832
	Female	43 (36.13)	147 (40.05)		190 (39.09)	25 (37.88)	69 (45.10)	
Age (years)		5.76 (±1.74)	5.63 (±1.68)	0.5024	5.66 (± 1.69)	5.44 (± 1.56)	5.46 (± 1.58)	0.2669
Bone age (years)		4.45 (±1.79)	4.22 (±1.64)	0.3002	4.27 (± 1.68)*	4.66 (± 1.59)*†	4.71 (± 1.75)†	0.0180
Height (cm)		104.37 (±9.71)	103.77 (±9.24)	0.5017	103.92 (± 9.35)	102.72 (± 9.84)	102.74 (± 9.15)	0.3034
Height SDS		-2.77 (±0.80)	-2.64 (±0.55)	0.2821	-2.69 (±0.70)	-2.75 (± 0.62)	-2.61 (± 0.47)	0.3691
Weight (kg)		17.53 (±4.00)	17.08 (±3.52)	0.4184	17.19 (± 3.64)*	16.58 (± 3.72)*†	15.96 (± 3.56)†	0.0014
Weight SDS		-1.93 (±1.09)	-1.96 (±0.88)	0.6473	-1.96 (± 0.94)*	-2.23 (± 0.84)*†	-2.45 (± 1.03)†	<0.0001
BMI (kg/m^2^)		16.00 (±1.68)	15.72 (±1.38)	0.2578	15.79 (± 1.47)*	15.41 (± 1.31)*†	14.92 (± 1.48)†	<0.0001
BMI SDS		-0.20 (±1.09)	-0.34 (±0.96)	0.2121	-0.30 (± 1.00)*	-0.55 (± 0.88)*†	-0.92 (± 1.08)†	<0.0001
IGF-I (ng/mL)		98.54 (±51.99)	116.80 (±55.98)	0.0121	112.38 (± 55.53)	116.29 (± 53.47)	131.89 (± 67.08)	0.0503
IGF-I SDS		-0.90 (±0.89)	-0.72 (±0.83)	0.0614	-0.76 (± 0.84)*	-0.73 (± 0.74)*†	-0.46 (± 0.97)†	0.0184
IGFBP-3 (ng/mL)		2278.40 (±926.70)	2596.16 (±989.30)	0.0318	2519.34 (± 982.36)	2360.86 (± 1002.02)	2701.26 (± 1033.86)	0.2581
IGFBP-3 SDS		-0.19 (±2.09)	0.44 (±2.14)	0.0339	0.29 (± 2.14)	-0.13 (± 2.05)	0.77 (± 2.36)	0.2030
Initial rhGH dose (mg/kg/week)		0.25 (±0.05)	0.25 (±0.05)	0.9821	0.25 (± 0.05)*	0.25 (± 0.06)*	0.27 (± 0.05)†	<0.0001
Peak GH level (ng/ml)		3.40 (±1.18)	7.70 (±1.41)	<0.0001	6.65 (± 2.29)*	20.26 (± 12.93)†	19.71 (± 12.11)†	<0.0001

BMI, body mass index; IGF-I, insulin-like growth factor-I; IGFBP-3, insulin-like growth factor-binding protein-3; SDS, standard deviation score; IGHD, idiopathic growth hormone deficiency; ISS, idiopathic short stature; SGA, small for gestational age.

Data are expressed as the mean ± standard deviation (SD) or number (%).

*^, †, ‡^, The same superscript indicates that there is no significant difference between the groups based on the Tukey multiple comparison test.

### Comparison of the change in height, serum IGF-I and IGFBP-3 levels

IGFBP-3 SDS was significantly reduced in CGHD group before rhGH treatment compared with the PGHD group; however, the height SDS, Δheight SDS, IGF-1 SDS, ΔIGF-1 SDS, and ΔIGFBP-3 SDS were not significantly different between them before and after rhGH treatment at 1 year and 2 years. A similar significant improvement in height SDS was observed throughout the treatment in the two groups (p < 0.0001) and the increase in IGF-I SDS and IGFBP-3 SDS also showed a similar pattern (Table 2).

**Table 2:** Height, serum IGF-I, and IGFBP-3 levels at the beginning of and during rhGH treatment in children with IGHD (CGHD vs PGHD).

	CGHD (n = 119)				PGHD (n = 367)				p-value (Intercomparison)
	n	mean (±SD)	median	(min, max)	n	mean (±SD)	median	(min, max)	
Height SDS									
Baseline	119	-2.82 (±0.82)	-2.56	(-8.10, -1.94)	367	-2.65 (±0.60)	-2.49	(-7.12, -1.85)	0.1815
1 year	115	-1.86 (±0.81)	-1.70	(-5.35, -0.51)	353	-1.80 (±0.68)	-1.70	(-6.64, -0.25)	0.6825
2 years	71	-1.45 (±0.77)	-1.42	(-3.45, -0.04)	241	-1.42 (±0.68)	-1.35	(-4.02, -0.25)	0.8652
ΔHeight SDS									
1 year—Baseline	115	0.93 (±0.43)	0.87	(0.14, 3.12)	353	0.85 (±0.32)	0.84	(0.08, 1.90)	0.1492
2 years—Baseline	71	1.39 (±0.64)	1.31	(0.14, 4.72)	241	1.23 (±0.38)	1.20	(0.02, 2.66)	0.0900
2 years—1 year	71	0.40 (±0.29)	0.41	(-0.29, 1.60)	234	0.38 (±0.22)	0.38	(-0.20, 1.11)	0.1234
p-value (intracomparison)^1^		<0.0001				<0.0001			
p-value (intracomparison)^2^		<0.0001				<0.0001			
p-value (intracomparison)^3^		<0.0001				<0.0001			
IGF-1 SDS									
Baseline	93	-0.90 (±0.89)	-0.96	(-3.21, 2.25)	288	-0.72 (±0.83)	-0.74	(-2.60, 3.34)	0.0614
1 year	82	0.71 (±1.69)	0.31	(-2.35, 8.48)	277	0.84 (±1.37)	0.54	(-1.70, 6.00)	0.1655
2 years	57	0.79 (±1.87)	0.37	(-2.13, 7.69)	182	1.13 (±1.53)	0.82	(-1.56, 7.16)	0.0595
ΔIGF-1 SDS									
1 year—Baseline	68	1.61 (±1.58)	1.56	(-3.49, 8.09)	238	1.55 (±1.24)	1.44	(-2.37, 5.62)	0.6331
2 years—Baseline	49	1.59 (±1.89)	1.28	(-4.38, 8.10)	159	1.82 (±1.64)	1.55	(-2.79, 7.63)	0.3585
2 years—1 year	46	-0.04 (±1.32)	-0.11	(-3.39, 3.03)	151	0.20 (±1.47)	0.06	(-4.08, 4.36)	0.4252
p-value (intracomparison)^1^		<0.0001				<0.0001			
p-value (intracomparison)^2^		<0.0001				<0.0001			
p-value (intracomparison)^3^		0.8327				0.1212			
IGFBP-3 SDS									
Baseline	66	-0.19 (±2.09)	-0.58	(-3.82, 4.80)	207	0.44 (±2.14)	0.05	(-3.29, 8.03)	0.0339
1 year	54	1.04 (±2.40)	0.84	(-2.57, 8.69)	194	1.50 (±2.60)	0.79	(-4.00, 9.12)	0.2631
2 years	41	1.19 (±2.50)	0.65	(-3.53, 6.62)	122	1.83 (±2.85)	1.29	(-2.98, 10.14)	0.2731
ΔIGFBP-3 SDS									
1 year—Baseline	48	0.92 (±1.61)	0.76	(-3.75, 4.69)	153	1.12 (±2.00)	1.07	(-4.63, 8.52)	0.5451
2 years—Baseline	34	1.02 (±2.19)	1.00	(-2.47, 6.40)	97	1.34 (±2.08)	0.91	(-3.92, 8.28)	0.4505
2 years—1 year	34	0.00 (±1.58)	-0.17	(-3.37, 3.05)	99	0.10 (±1.82)	0.14	(-5.64, 5.30)	0.5421
p-value (intracomparison)^1^		0.0002				<0.0001			
p-value (intracomparison)^2^		0.0105				<0.0001			
p-value (intracomparison)^3^		0.9912				0.4749			

BMI, body mass index; IGF-I, insulin-like growth factor-I; IGFBP-3, insulin-like growth factor-binding protein-3; SDS, standard deviation score; IGHD, idiopathic growth hormone deficiency; ISS, idiopathic short stature; SGA, small for gestational age.

Data are expressed as the mean ± standard deviation (SD) or number (%).

*^, †, ‡^, The same superscript indicates that there is no significant difference between the groups based on the Tukey multiple comparison test.

^1^, 1 year versus baseline;

^2^, 2 years versus baseline;

^3^, 2 years versus 1 year.

Before rhGH treatment, the mean ± SD for height SDS was -2.69 ± 0.70 (range: -8.10, -1.85) in the IGHD group, -2.75 ± 0.62 (range: -4.73, -1.91) in the ISS group, and -2.61 ± 0.47 (range: -4.03, -1.93) in the SGA group. A similar significant improvement in height SDS was detected throughout the treatment in all three groups (p < 0.0001). During the 2-year treatment, Δheight SDS was the highest in the IGHD group compared to that in the other two groups despite no statistical difference. IGF-I SDS was significantly different among the three groups (p = 0.0184 at the baseline, p = 0.0059 after 1 year of treatment, and p = 0.0156 after 2 years of treatment). However, ΔIGF-I, IGFBP-3 SDS and ΔIGFBP-3 SDS were not significantly different among the three groups at the baseline, and after 1 and 2 years of treatment. IGF-I SDS and IGFBP-3 SDS increased after rhGH therapy after 1 and 2 years of treatment in all three groups. ΔIGF-1, and ΔIGFBP-3 during 1 and 2 years of treatment, were significantly increased compared to those at the baseline (p < 0.05) while ΔIGF-1 and ΔIGFBP-3 were similar between 1 and 2 years of treatment time points (p > 0.05) (Table 3).

**Table 3:** Height, serum IGF-I and IGFBP-3 levels at the beginning of and during rhGH treatment in children with IGHD, ISS, and SGA.

	IGHD (n = 486)				ISS (n = 66)				SGA (n = 153)				p-value (Intercomparison)
	n	mean (±SD)	median	(min, max)	n	mean (±SD)	median	(min, max)	n	mean (±SD)	median	(min, max)	
Height SDS													
Baseline	486	-2.69 (±0.70)	-2.51	(-8.10, -1.85)	66	-2.75 (±0.62)	-2.65	(-4.73, -1.91)	153	-2.61 (±0.47)	-2.51	(-4.03, -1.93)	0.3691
1 year	468	-1.81 (±0.71)	-1.70	(-6.64, -0.25)	64	-1.93 (±0.60)	-1.85	(-3.77, -0.72)	150	-1.81 (±0.62)	-1.67	(-3.54, -0.38)	0.1697
2 years	312	-1.43 (±0.70)	-1.36	(-4.02, 0.25)	33	-1.62 (±0.65)	-1.49	(-3.35, -0.33)	103	-1.39 (±0.65)	-1.27	(-3.27, -0.26)	0.1666
ΔHeight SDS													
1 year—Baseline	468	0.87 (±0.35)	0.85	(0.08, 3.12)	64	0.80 (±0.30)	0.77	(0.18, 1.48)	150	0.80 (±0.32)	0.80	(0.02, 1.99)	0.0663
2 years—Baseline	312	1.27 (±0.46)	1.23	(0.02, 4.72)	33	1.21 (±0.42)	1.12	(0.38, 2.14)	103	1.23 (±0.40)	1.20	(0.29, 2.26)	0.6007
2 years—1 year	305	0.39 (±0.24)	0.39	(-0.29, 1.60)	33	0.36 (±0.24)	0.35	(-0.09, 1.05)	101	0.43 (±0.23)	0.40	(-0.17, 1.13)	0.1862
p-value (intracomparison)^1^		<0.0001				<0.0001				<0.0001			
p-value (intracomparison)^2^		<0.0001				<0.0001				<0.0001			
p-value (intracomparison)^3^		<0.0001				<0.0001				<0.0001			
IGF-1 SDS													
Baseline	380	-0.76 (±0.84)*	-0.84	(-3.21, 3.34)	44	-0.73 (±0.74) *†	-0.83	(-2.09, 1.22)	110	-0.46 (±0.97) †	-0.60	(-2.13, 4.99)	0.0184
1 year	359	0.81 (±1.45)*	0.50	(-2.35, 8.48)	45	0.58 (±1.19)*	0.48	(-1.69, 2.83)	125	1.18 (±1.38) †	0.96	(-1.18, 6.24)	0.0059
2 years	239	1.04 (±1.62)*	0.70	(-2.13, 7.69)	24	0.64 (±1.39)*	0.56	(-1.56, 5.02)	82	1.37 (±1.25) †	1.34	(-0.81, 4.53)	0.0156
ΔIGF-1 SDS													
1 year—Baseline	306	1.56 (±1.32)	1.47	(-3.49, 8.09)	31	1.44 (±0.93)	1.33	(-0.11, 3.28)	99	1.61 (±1.13)	1.54	(-0.52, 5.28)	0.8917
2 years—Baseline	208	1.77 (±1.70)	1.47	(-4.38, 8.10)	19	1.44 (±1.50)	1.03	(-0.67, 5.82)	62	1.85 (±1.32)	1.81	(-2.01, 4.88)	0.1936
2 years—1 year	197	0.14 (±1.44)	0.04	(-4.08, 4.36)	21	-0.00 (±1.21)	-0.35	(-1.89, 3.31)	77	0.04 (±1.29)	0.09	(-2.83, 3.45)	0.6984
p-value (intracomparison)^1^		<0.0001				<0.0001				<0.0001			
p-value (intracomparison)^2^		<0.0001				<0.0001				<0.0001			
p-value (intracomparison)^3^		0.1652				0.9965				0.8004			
IGFBP-3 SDS													
Baseline	273	0.29 (±2.14)	-0.17	(-3.82, 8.03)	19	-0.13 (±2.05)	-0.87	(-2.77, 5.82)	64	0.77 (±2.36)	0.31	(-3.61, 8.13)	0.2030
1 year	248	1.40 (±2.56)	0.8	(-4.00, 9.12)	32	0.92 (±2.55)	0.15	(-3.01, 6.00)	79	1.58 (±3.14)	0.05	(-2.65, 11.72)	0.5977
2 years	163	1.67 (±2.77)	1.04	(-3.53, 10.14)	16	1.28 (±3.41)	0.63	(-3.34, 6.69)	59	1.64 (±2.67)	0.98	(-2.02, 7.58)	0.7184
ΔIGFBP-3 SDS													
1 year—Baseline	201	1.07 (±1.91)	0.95	(-4.63, 8.52)	15	1.24 (±1.61)	1.35	(-0.95, 4.53)	55	1.09 (±1.84)	0.79	(-1.76, 6.32)	0.8140
2 years—Baseline	131	1.25 (±2.11)	0.94	(-3.92, 8.28)	7	0.43 (±1.99)	0.93	(-3.44, 2.35)	39	0.99 (±1.71)	1.00	(-2.06, 5.80)	0.4793
2 years—1 year	133	0.07 (±1.76)	0.01	(-5.64, 5.30)	14	-0.18 (±2.14)	-0.39	(-2.79, 4.20)	54	0.33 (±2.12)	0.38	(-4.96, 6.29)	0.2472
p-value (intracomparison)^1^		<0.0001				0.0098				<0.0001			
p-value (intracomparison)^2^		<0.0001				0.5870				0.0009			
p-value (intracomparison)^3^		0.6542				0.7579				0.0655			

BMI, body mass index; IGF-I, insulin-like growth factor-I; IGFBP-3, insulin-like growth factor-binding protein-3; SDS, standard deviation score; IGHD, idiopathic growth hormone deficiency; ISS, idiopathic short stature; SGA, small for gestational age.

Data are expressed as the mean ± standard deviation (SD) or number (%).

*^, †, ‡^, The same superscript indicates that there is no significant difference between the groups based on the Tukey multiple comparison test.

^1^, 1 year versus baseline;

^2^, 2 years versus baseline;

^3^, 2 years versus 1 year.

### Comparison of the growth response after 1 year of rhGH therapy with clinical parameters and IGF-I/IGFBP-3 serum levels at the baseline

When biochemical data were analyzed according to good or poor 1-year Δheight, baseline BMI was lower in poor response than in good response CGHD group (p = 0.0327). Age (p = 0.0003) and BA (p = 0.0004) at the beginning of the rhGH treatment were higher in poor response group than in good response PGHD group. Sex, MPH, height SDS, IGF-I, and IGFBP-3 level/SDS were similar in both the groups (Table 4).

**Table 4:** Characteristics of patients with good and poor response to rhGH therapy after 1 year in IGHD.

		CGHD			PGHD		
		Poor response	Good response	p-value (Intercomparison)	Poor response	Good response	p-value (Intercomparison)
		n = 10	n = 105		n = 39	n = 314	
Sex, n (%)	Male	6 (60.00)	68 (64.76)	0.7426	25 (64.10)	186 (59.24)	0.5588
	Female	4 (40.00)	37 (35.24)		14 (35.90)	128 (40.76)	
MPH SDS	n	8	100		38	297	
	mean (±SD)	-0.82 (±0.55)	-0.85 (±0.75)	0.9766	-1.21 (±0.92)	-0.89 (±0.72)	0.0651
Height SDS	n	9	101		37	300	
	mean (±SD)	-2.83 (±0.80)	-2.73 (±0.79)	0.7360	-2.68 (±0.69)	-2.63 (±0.53)	0.6506
BMI SDS	n	8	100		37	290	
	mean (±SD)	-0.86 (±1.71)	-0.17 (±0.98)	0.0327	-0.43 (±1.06)	-0.34 (±0.96)	0.5976
Age (years)	n	10	105		39	314	
	mean (±SD)	5.90 (±2.33)	5.78 (±1.66)	0.8678	6.62 (±1.68)	5.51 (±1.64)	0.0003
Bone age (years)	n	7	96		35	291	
	mean (±SD)	5.30 (±1.26)	4.41 (±1.82)	0.1535	5.18 (±1.68)	4.11 (±1.61)	0.0004
IGF-1 (ng/mL)	n	8	81		32	247	
	mean (±SD)	108.53 (±42.48)	98.16 (±52.97)	0.3801	121.76 (±58.64)	117.48 (±55.97)	0.8016
IGF-1 SDS	n	8	81		32	247	
	mean (±SD)	-0.95 (±0.37)	-0.88 (±0.90)	0.9373	-0.80 (±0.92)	-0.69 (±0.81)	0.3603
IGFBP-3 (ng/mL)	n	6	57		25	173	
	mean (±SD)	2,606.70 (±751.93)	2,252.34 (±935.69)	0.2557	2,509.63 (±1,067.13)	2,614.17 (±987.25)	0.5287
IGFBP-3 SDS	n	6	57		25	173	
	mean (±SD)	0.26 (±1.82)	-0.22 (±2.10)	0.4358	0.07 (±2.38)	0.50 (±2.12)	0.3119

BMI, body mass index; IGF-I, insulin-like growth factor-I; IGFBP-3, insulin-like growth factor-binding protein-3; MPH, mid-parental height; SDS, standard deviation score; IGHD, idiopathic growth hormone deficiency; ISS, idiopathic short stature; SGA, small for gestational age.

Data are expressed as the mean ± standard deviation (SD) or number (%).

*^, †, ‡^, The same superscript indicates that there is no significant difference between the groups based on the Tukey multiple comparison test.

Poor response: ΔHeight SDS in 1st year < 0.46 (IGHD), ΔHeight SDS in 1st year < 0.3 (ISS), ΔHeight SDS in 1st year < 0.34 (SGA).

Good response: ΔHeight SDS in 1st year ≥ 0.46 (IGHD), ΔHeight SDS in 1st year ≥ 0.3 (ISS), ΔHeight SDS in 1st year ≥ 0.34 (SGA).

In comparison IGHD, SGA and SGA groups, we found that the age (p = 0.0010) and BA (p = 0.0002) at the beginning of the rhGH therapy were significantly higher in children with a poor response than in those with a good response in the IGHD group. There was no difference in sex, MPH SDS, BMI SDS, serum IGF-1 and IGFBP-3 levels in the IGHD group. ISS and SGA groups showed no significant difference in sex, MPH SDS, height SDS, BMI SDS, age, BA, IGF-1 and IGFBP-3 between poor and good responder groups (Table 5).

**Table 5:** Characteristics of patients with good and poor response to rhGH therapy after 1 year.

		IGHD			ISS			SGA		
		Poor response	Good response	p-value (Intercomparison)	Poor response	Good response	p-value (Intercomparison)	Poor response	Good response	p-value (Intercomparison)
		n = 49	n = 419		n = 3	n = 61		n = 10	n = 140	
Sex, n (%)	Male	31 (63.27)	254 (60.62)	0.7196	3 (100.00)	38 (62.30)	0.5473	7 (70.00)	75 (53.57)	0.3493
	Female	18 (36.73)	165 (39.38)		0 (0.00)	23 (37.70)		3 (30.00)	65 (46.43)	
MPH SDS	n	46	397		2	59		8	128	
	mean (±SD)	-1.15 (±0.87)	-0.88 (±0.73)	0.0738	-0.63 (±0.13)	-1.07 (±0.74)	0.4119	-1.01 (±0.79)	-0.94 (±0.68)	0.7888
Height SDS	n	46	401		3	58		9	137	
	mean (±SD)	-2.71 (±0.70)	-2.65 (±0.61)	0.8064	-2.54 (±0.15)	-2.74 (±0.63)	0.5616	-2.83 (±0.43)	-2.60 (±0.47)	0.0912
BMI SDS	n	45	390		3	54		8	133	
	mean (±SD)	-0.50 (±1.19)	-0.29 (±0.97)	0.2584	-1.19 (±0.86)	-0.49 (±0.86)	0.1773	-0.63 (±1.32)	-0.94 (±1.07)	0.4322
Age (years)	n	49	419		3	61		10	140	
	mean (±SD)	6.47 (±1.83)	5.58 (±1.65)	0.0010	6.33 (±2.08)	5.43 (±1.55)	0.4190	5.70 (±1.70)	5.44 (±1.59)	0.7027
Bone age (years)	n	42	387		3	53		8	120	
	mean (±SD)	5.20 (±1.60)	4.19 (±1.67)	0.0002	4.00 (±0.50)	4.70 (±1.64)	0.4648	5.08 (±2.37)	4.68 (±1.73)	0.8017
IGF-1 (ng/mL)	n	40	328		2	41		6	101	
	mean (±SD)	119.11 (±55.55)	112.71 (±55.79)	0.4988	159.50 (±61.52)	115.17 (±53.32)	0.2595	128.67 (±51.17)	133.79 (±68.30)	0.9247
IGF-1 SDS	n	40	328		2	41		6	101	
	mean (±SD)	-0.83 (±0.84)	-0.74 (±0.84)	0.3949	0.12 (±0.34)	-0.76 (±0.74)	0.1052	-0.42 (±0.87)	-0.45 (±0.98)	0.7561
IGFBP-3 (ng/mL)	n	31	230		1	18		4	59	
	mean (±SD)	2,528.42 (±1,003.37)	2,524.50 (±985.23)	0.9949	5,480.00	2,187.58 (±677.50)	0.1381	2,747.00 (±866.28)	2,700.89 (±1,059.06)	0.8995
IGFBP-3 SDS	n	31	230		1	18		4	59	
	mean (±SD)	0.11(±2.25)	0.32(±2.13)	0.6020	5.82	-0.46(±1.51)	0.1381	0.81 (±1.68)	0.78(±2.43)	0.7049

BMI, body mass index; IGF-I, insulin-like growth factor-I; IGFBP-3, insulin-like growth factor-binding protein-3; MPH, mid-parental height; SDS, standard deviation score; IGHD, idiopathic growth hormone deficiency; ISS, idiopathic short stature; SGA, small for gestational age.

Data are expressed as the mean ± standard deviation (SD) or number (%).

*^, †, ‡^, The same superscript indicates that there is no significant difference between the groups based on the Tukey multiple comparison test.

Poor response: ΔHeight SDS in 1st year < 0.46 (IGHD), ΔHeight SDS in 1st year < 0.3 (ISS), ΔHeight SDS in 1st year < 0.34 (SGA).

Good response: ΔHeight SDS in 1st year ≥ 0.46 (IGHD), ΔHeight SDS in 1st year ≥ 0.3 (ISS), ΔHeight SDS in 1st year ≥ 0.34 (SGA).

### Correlation between Δheight SDS and serum ΔIGF-I and ΔIGFBP-3 levels

A positive correlation was observed between Δheight SDS and ΔIGF-I (r = 0.1331, p = 0.0225), ΔIGF-I SDS (r = 0.1539, p = 0.0082), ΔIGFBP 3 (r = 0.1898, p-0.0348), and ΔIGFBP-3 SDS (r = 0.2145, p-0.0167) during 1 year of treatment in the IGHD group. However, ISS and SGA groups showed no correlation between Δheight and serum ΔIGF-I and ΔIGFBP-3 levels during 1 and 2 years of therapy (Table 6).

**Table 6:** Correlation analysis between Δheight SDS and ΔIGF-I or ΔIGFBP-3 during the 1^st^ and 2^nd^ year of rhGH therapy.

	IGHD			ISS			SGA		
	n	γ	p-value	n	γ	p-value	n	γ	p-value
	Δ Height SDS (1 years—Baseline)								
ΔIGF-1	294	0.1331	0.0225	30	-0.0319	0.8669	96	-0.0373	0.7182
ΔIGF-1 SDS	294	0.1539	0.0082	30	-0.0906	0.6339	96	-0.0958	0.3529
ΔIGFBP-3	190	-0.0037	0.9598	15	-0.012	0.9662	54	-0.1071	0.4408
ΔIGFBP-3 SDS	190	0.0252	0.7298	15	0.0902	0.7491	54	-0.081	0.5604
	Δ Height SDS (2 years—Baseline)								
ΔIGF-1	202	0.0647	0.3600	14	0.4564	0.1009	62	0.1242	0.3360
ΔIGF-1 SDS	202	0.1196	0.0900	14	0.3934	0.1640	62	-0.0140	0.9140
ΔIGFBP-3	124	0.1898	0.0348	5	0.2184	0.7241	39	0.0409	0.8049
ΔIGFBP-3 SDS	124	0.2145	0.0167	5	0.2353	0.7032	39	-0.0124	0.9405

IGF-I, insulin-like growth factor-I; IGFBP-3, insulin-like growth factor-binding protein-3; SDS, standard deviation score; IGHD, idiopathic growth hormone deficiency; ISS, idiopathic short stature; SGA, small for gestational age.

ΔIGF-I SDS (beta = 0.0413, p = 0.0322) and ΔIGFBP-3 SDS (beta = 0.0352, p = 0.0467) were significantly positively related to Δheight SDS during 2 years of therapy, revealed using ANCONA analysis, after adjusting for baseline height as covariates in the IGHD group, which was not observed in ISS and SGA groups (Table 7).

**Table 7:** Analysis of covariance (ANCOVA) of Δheight SDS during the 2^nd^ year of rhGH therapy using the ΔIGF-I/ΔIGFBP-3 ratio as a fixed factor and height at the baseline as a covariate.

Parameter (2 years—Baseline)	Δ Height SDS (2 years—Baseline)								
	IGHD			ISS			SGA		
	n	beta	p-value	n	beta	p-value	n	beta	p-value
Intercept	202	0.5867	<0.0001	14	0.6976	0.2091	62	1.2946	<0.0001
Height SDS (Baseline)	202	0.2182	<0.0001	14	0.1862	0.2962	62	0.0168	0.8857
ΔIGF-1 SDS	202	0.0413	0.0322	14	0.1074	0.3279	62	0.0037	0.9268
Intercept	124	1.2880	<0.0001	5	1.1049	0.1595	39	1.1211	0.0073
Height SDS (Baseline)	124	0.0345	0.5334	5	0.0239	0.8905	39	0.0334	0.8238
ΔIGFBP-3 SDS	124	0.0352	0.0467	5	0.0274	0.7419	39	0.0031	0.9423

IGF-I, insulin-like growth factor-I; IGFBP-3, insulin-like growth factor-binding protein-3; SDS, standard deviation score; IGHD, idiopathic growth hormone deficiency; ISS, idiopathic short stature; SGA, small for gestational age.

## Discussion

In this study, we demonstrated the efficacy of serum IGF-I and IGFBP-3 levels in adequately reflecting the height outcome of the first 2 years of rhGH therapy in prepubertal short stature children in IGHD, ISS, and SGA groups. We found that the response to rhGH therapy depended on ΔIGF-I and ΔIGFBP-3 levels in the IGHD group. Indicating that the continuous increase in height for GHD groups in the 2^nd^ year of treatment was related to the increased concentration of IGF-I and IGFBP-3 in the serum.

Growth response in the first year of rhGH therapy is one of the best indicators of long-term stature growth [25, 26]. Initial growth rate, bone remodeling marker levels after 3 months of rhGH therapy, and height SDS have been used to evaluate response to treatment [27, 28]. We used height SDS as an indicator and found that children presented with a good Δheight SDS after 1 year of therapy in the IGHD group. The PGHD group had a lower CA and BA at the beginning of the rhGH therapy, showing the importance of early diagnosis and rhGH therapy in IGHD. The patterns of growth and increase in IGF-I and IGFBP-3 levels were similar between CGHD and PGHD subjects. In this study, MPH was not different between CGHD and PGHD and among IGHD, ISS, and SGA groups. Thus, judging the growth responsiveness by clinical data such as MPH alone is insufficient; instead, genetic testing results are more efficient in the prediction of rhGH therapy response in short stature children, especially in no-growth hormone deficiency [29].

The GH-IGF-1 axis is the primary key to the endocrine system that controls linear growth during childhood [30]. GH regulates IGF-I release by acting on the liver, which modulates bone length during childhood [7, 31]. IGF-I secretion does not depend on diurnal variation or pulsatility [6]. IGF-I binds to specific IGFBPs in the circulation. IGFBP-3 is the main abundant circulating IGFBP and plays an important role in the metabolic effect of IGF-1 [32]. IGFBP-3 inhibits the bioactivity of IGF-I through binding, thereby reducing the concentration of free IGF-I in the circulation [32]. IGFBP-3 forms a ternary complex with IGF-I through its acid-labile subunits, and the formation of this complex extends their half-life in the circulation [30]. The half-life of IGF-I is about 10 min in free form, and 12–15 h in the ternary complex form [33].

There are continuing efforts to find markers that reflect growth effects. In recent years, references by age, gender, and diagnosis have been derived from many rhGH-treated prepubertal children registries such as the National Cooperative Growth Study (n = 7000) and Pfizer International Growth Database (KIGS) (n = 8500) [21, 34]. Although the criteria for patients with IGHD are slightly different in each registry, it is commonly reported that the growth rate in the first year is a future growth response parameter, and the growth rate decreases during the second year. Similar results have been reported from 162 prepubertal Swedish children with IGHD and SGA [35]. As a related study, recently Cho, et al. [36] reported a prediction analysis of first-year growth in response to rhGH therapy in 345 prepubertal Korean children with IGHD in the same LGS registry as this study. Therefore, in this study, we focused on the relationship between IGF-I and IGFBP-3 based on growth rates in the first and second years.

We presented IGF-I and IGFBP-3 as growth markers. IGF-1 receptor (IGF-IR) dysfunctions and a reduced number of IGF-IR via gene mutations could lead to IGF-I insensitivity [37]. Single nucleotide polymorphisms in the IGFBP-3-coding region are associated with height variation [37]. Especially, the A allele in the IGFBP-3 promoter region reportedly causes an increase in IGFBP-3 expression, which reflects the growth rate in rhGH therapy in prepubertal children with GHD [38] and Turner syndrome [39], but not ISS [40].

GH binds to the GH receptor and stimulates STAT5b activation in the hepatocytes [27]. Subsequently, it stimulates the production and secretion of not only IGF-I but also IGFBP-3 from the liver [7]. Serum levels of IGF-I and IGFBP-3 are even more important in GH/IGF-I assessments for older children and children younger than 3 years of age [7]. However, many hormones control circulating IGF-1 levels, such as insulin [41], thyroid hormone [42], and sex hormones [43]. Especially, low levels of estrogen stimulate IGF-1 secretion, but high levels of it decrease IGF-1 secretion [43]. In our study, the subjects did not have malnutrition, chronic endocrine/nutritional/inflammatory diseases, and prepuberty.

Circulating IGF-I helps monitor compliance and efficacy in response to rhGH therapy despite the limitations of decreased IGF-I concentration in a catabolic state and within-individual and inter-assay variability [6], but there is no clarity on the effect of the IGF-I/IGFBP-3 molar ratio [2, 5, 25]. In our study, the increase in height continued to improve over 2 years of rhGH therapy in IGHD, ISS, and SGA groups, while IGF-I SDS and IGFBP-3 SDS reached a plateau for all three groups. Domene et al. [44] showed that an increase in serum IGF-I levels over IGFBP-3 levels in patients with GHD was more pronounced compared to that in the SGA group perhaps due to differences in IGF-I levels and/or GH susceptibility at the start of the treatment. Our results also supported their results.

Low ΔIGF-I levels during the first 3 months of therapy in non-IGHD short stature subjects can be an indicator of GH resistance and predict the response to rhGH therapy [27]. Our results confirmed this only for the IGHD group. Previous reports showed that ΔIGF-I is approximately 180 ng/mL in healthy prepubertal short children [45] and under 120 ng/mL in GH-resistant children during the 1^st^ year of therapy [46].

There is no consensus on the usefulness of serum IGFBP-3 levels due to the lack of related data in rhGH therapy. Furthermore, IGFBP-3 is fragmented by proteases after being secreted [2] under certain conditions, such as inflammation [47]. Moreover, fragmented IGFBP-3 has no function [48, 49]. These increase the doubt of using serum IGFBP-3 levels as a tool to monitor rhGH therapy efficacy. Recently, Perez-Colon et al. [27] reported the baseline serum IGFBP-3 level as the key to determining not only the degree of GH resistance but also the growth response to rhGH and IGF-1 treatment in non-GH-deficient short stature and IGF-1 deficient children. Seino et al. [28] reported that baseline IGFBP-3 is significantly related to height velocity. Their data showed a major role of IGFBP-3 in response to rhGH treatment. In the present study, both baseline IGF-I SDS and IGFBP-3 SDS were not notably different between children who showed poor or good height response to rhGH therapy after 1 year in each group. Also, despite a significant increase in height, serum ΔIGFBP-3 levels among the three groups showed a different increasing rate. Therefore, we analyzed serum ΔIGFBP-3 during the 1^st^ and 2^nd^ years of thGH therapy and found that ΔIGFBP-3 levels were positively related with Δheight SDS in rhGH therapy in the IGHD group. To the best of our knowledge, this study is the first to report such results. Studies on the predictiveness of serum IGFBP-3 levels in rhGH therapy are limited. Following rhGH therapy, higher serum IGF-I levels, than IGFBP-3, have been reported [27], which suggests that GH has different effects on IGF-I and IGFBP-3 [50] despite the changes in serum IGFBP-3 levels, for example, serum IGF-I levels are correlated with GH concentration. Moreover, IGFBP-3 is reportedly involved in apoptosis and growth inhibition of cancer cells [6]. Therefore, more research is warranted to clarify the function of IGFBP-3 in response to GH.

Our study has several limitations. First, the number of subjects evaluated for the 2-year rhGH therapy was relatively small, especially for ISS and SGA groups, and the study period was short, with just two years. Retrospective studies after reaching adult height are needed to verify that adult height predictions based on growth and hormonal responses during the first two years are useful and accurate. Second, we did not assess serum insulin, IGF-II, free IGF-I, PAPP-A2, and intact IGFBP-3 levels. Third, although children who had already been diagnosed with genetic abnormalities were excluded, not all patients underwent genetic testing for short stature. As the height of children is inherited from the parents in a polygenic manner, a genetic analysis would be needed to find a good responder group, especially in ISS and SGA groups [29]. Fourth, we collected the clinical and laboratory data such as IGF-I and IGFBP3 levels from multiple centers (total 73 sites). As IGF-I and IGFBP-3 levels were measured at different institutions, this could contribute to a measurement bias. However, IGF-I and IGFBP-3 measurements were higher standardized, which were converted to SDS values by LGS [37]. Fifth, all the subjects were not tested using the same GH stimulation test. As mentioned, we determined the peak GH levels from two separate GH stimulation test results using insulin, clonidine, L-arginine, L-dopa, or glucagon. However, the provocation test using insulin was common among all of them.

Despite its limitation, this study has several important advantages. First, the large sample size with homogeneous subjects such as prepubertal children and a single ethnic group race in LGS data were analyzed in this study. Additionally, the LGS data are significant because they can reduce errors among researchers when the information is collected through multicenter databases [19]. Second, various clinical parameters were positively analyzed and subdivided among patients classified as IGHD, especially CGHD and PGHD, and ISS and SGA. Third, although a few previous studies about IGF-I/IGFBP-3 and growth response consisting of GHD, ISS, and SGA in patients exist, there has been no study involving a relatively large cohort of prepubertal GHD patients like ours using our rhGH therapy.

In summary, most prepubertal children included in this study, who received rhGH therapy, showed an increase in height after the 1^st^ and 2^nd^ year of rhGH treatment in all three groups. The subjects with IGHD had normalized serum IGF-I values within the 1st year of rhGH therapy; serum IGF-I and IGFBP-3 levels were associated with height improvement.

In conclusion, IGF-I and IGFBP-3 are the primary factors involved in growth; therefore, it is important to identify more factors that affect their levels in serum. We believe that serum ΔIGFBP-3 level may serve as a marker to predict the height response along with serum ΔIGF-I level in patients with IGHD. Also, our results may help improve the overall growth outcomes of children receiving personalized rhGH therapy.

## References

[1] Rhie YJ, Yoo JH, Choi JH, Chae HW, Kim JH, Chung S, et al. Long-term safety and effectiveness of growth hormone therapy in Korean children with growth disorders: 5-year results of LG Growth Study. PLoS One. 2019;14:e0216927. doi: 10.1371/journal.pone.0216927 31095622 PMC6522217

[2] Ballerini MG, Braslavsky D, Scaglia PA, Keselman A, Rodríguez ME, Martínez A, et al. Circulating IGF-I, IGFBP-3 and the IGF-I/IGFBP-3 Molar Ratio Concentration and Height Outcome in Prepubertal Short Children on rhGH Treatment over Two Years of Therapy. Horm Res Paediatr. 2017;88:354–363. doi: 10.1159/000479691 28926833

[3] Frystyk J. Utility of Free IGF-I Measurements Pituitary (2007) 10:181–187. doi: 10.1007/s11102-007-0025-y 17429595

[4] Sıklar Z, Öcal G, Berberoğlu M, Bilir P. Combined evaluation of IGF-1 and IGFBP-3 as an index of efficacy and safety in growth hormone treated patients. J Clin Res Pediatr Endocrinol. 2009;1:240–243. doi: 10.4274/jcrpe.v1i5.240 21274301 PMC3005747

[5] Bang P, Bjerknes R, Dahlgren J, Dunkel L, Gustafsson J, Juul A, et al. A comparison of different definitions of growth response in short prepubertal children treated with growth hormone. Horm Res Paediatr. 2011;75:335–345. doi: 10.1159/000322878 21228552

[6] Johannsson G, Bidlingmaier M, Biller BMK, Boguszewski M, Casanueva FF, Chanson P, et al. Growth Hormone Research Society perspective on biomarkers of GH action in children and adults. Endocr Connect. 2018;7:R126–R134. doi: 10.1530/EC-18-0047 29483159 PMC5868631

[7] Polidori N, Castorani V, Mohn A, Chiarelli F. Deciphering short stature in children. Ann Pediatr Endocrinol Metab. 2020;25:69–79. doi: 10.6065/apem.2040064.032 32615685 PMC7336267

[8] Sizonenko PC, Clayton PE, Cohen P, Hintz RL, Tanaka T, Laron Z. Diagnosis and management of growth hormone deficiency in childhood and adolescence. Part 1: diagnosis of growth hormone deficiency. Growth Horm IGF Res. 2001;11:137–165. doi: 10.1054/ghir.2001.0203 11735230

[9] Jin L, Shen F, Weinfeld M, Sergi C. Insulin growth factor binding protein 7 (IGFBP7)-related cancer and IGFBP3 and IGFBP7 crosstalk. Front Oncol. 2020;10:727. doi: 10.3389/fonc.2020.00727 32500027 PMC7242731

[10] Dauber A, Muñoz-Calvo MT, Barrios V, Domené HM, Kloverpris S, Serra-Juhé C, et al. Mutations in pregnancy-associated plasma protein A2 cause short stature due to low IGF-I availability. EMBO Mol Med. 2016;8:363–374. doi: 10.15252/emmm.201506106 26902202 PMC4818753

[11] Fujimoto M, Khoury JC, Khoury JC, Kalra B, Kumar A, Sluss P, et al. Anthropometric and biochemical correlates of PAPP-A2, free IGF-I, and IGFBP-3 in childhood. Eur J Endocrinol. 2020;182(3):363–374. doi: 10.1530/EJE-19-0859 31961798 PMC7238294

[12] Juul A, Bang P, Hertel NT, Main K, Dalgaard P, Jørgensen K, et al. Serum insulin-like growth factor-I in 1,030 healthy children, adolescents, and adults: relation to age, sex, stage of puberty, testicular size, and body mass index. J Clin Endocrinol Metab. 1994;78:744–752. doi: 10.1210/jcem.78.3.8126152 8126152

[13] Choi YJ, Lee YJ, Lee NY, Lee S-H, Kim S-K, Ahn M-B, et al. Discriminatory performance of insulin-like growth factor 1 and insulin-like growth factor binding protein-3 by correlating values to chronological age, bone age, and pubertal status for diagnosis of isolated growth hormone deficiency. Ann Pediatr Endocrinol Metab. 2020;25:240–247. doi: 10.6065/apem.2040018.009 32871649 PMC7788340

[14] Bayley N, Pinneau SR. Tables for predicting adult height from skeletal age: revised for use with the Greulich-Pyle hand standards. J Pediatr. 1952;40:423–441. doi: 10.1016/s0022-3476(52)80205-7 14918032

[15] Chung S, Yoo JH, Choi JH, Rhie YJ, Chae HW, Kim JH, et al. Design of the long-term observational cohort study with recombinant human growth hormone in Korean children: LG Growth Study. Ann Pediatr Endocrinol Metab. 2018;23:43–50. doi: 10.6065/apem.2018.23.1.43 29609449 PMC5894560

[16] Kim JH, Yun S, Hwang S, Shim JO, Chae HW, Lee YJ, et al. The 2017 Korean national growth charts for children and adolescents: development, improvement, and prospects. Korean J Pediatr. 2018;61:135–149. doi: 10.3345/kjp.2018.61.5.135 29853938 PMC5976563

[17] Mauras N, Walton P, Nicar M, Welch S, Rogol AD. Growth Hormone Stimulation Testing in Both Short and Normal Statured Children: Use of an Immunofunctional Assay. Pediatr Res. 2000;48:614–618. doi: 10.1203/00006450-200011000-00010 11044480

[18] Nam H-K, Kim HR, Lee K-H, Rhie Y-J. Idiopathic Short Stature Phenotypes among Korean Children: Cluster Analysis. Tohoku J Exp Med. 2019;248:193–200. doi: 10.1620/tjem.248.193 31353328

[19] Kang MJ, Kim EY, Shim YS, Jeong HR, Lee HJ, Yang S, et al. Factors affecting bone age maturation during 3 years of growth hormone treatment in patients with idiopathic growth hormone deficiency and idiopathic short stature: Analysis of data from the LG growth study. Medicine (Baltimore). 2019;98:e14962. doi: 10.1097/MD.0000000000014962 30946320 PMC6456092

[20] Smyczyńska J, Lewiński A, Hilczer M, Stawerska R, Karasek M. Partial growth hormone deficiency (GHD) in children has more similarities to idiopathic short stature than to severe GHD. Endokrynol Pol. 2007;58(3):182–187. 17940982

[21] Ranke MB, Lindberg A, on behalf of the KIGS International Board. Observed and predicted growth responses in prepubertal children with growth disorders: Guidance of growth hormone treatment by empirical variables. J Clin Endocrinol Metabol. 2010;95(3):1229–1237. doi: 10.1210/jc.2009-1471 20097713

[22] Boguszewski M, Jansson C, Rosberg S, Albertsson-Wikland K. Changes in serum insulin-like growth factor I (IGF-I) and IGF-binding protein-3 levels during growth hormone treatment in prepubertal short children born small for gestational age. J Clin Endocrinol Metab. 1996;81:3902–3908. doi: 10.1210/jcem.81.11.8923836 8923836

[23] Marshall WA, Tanner JM. Variations in the pattern of pubertal changes in boys. Arch Dis Child. 1970;45:13–23. doi: 10.1136/adc.45.239.13 5440182 PMC2020414

[24] Hyun SE, Lee BC, Suh BK, Chung SC, Ko CW, Kim HS, et al. Reference values for serum levels of insulin-like growth factor-I and insulin-like growth factor binding protein-3 in Korean children and adolescents. Clin Biochem. 2012;45:16–21. doi: 10.1016/j.clinbiochem.2011.10.003 22032863

[25] Murray PG, Dattani MT, Clayton PE. Controversies in the diagnosis and management of growth hormone deficiency in childhood and adolescence. Arch Dis Child. 2015;101:96–100. doi: 10.1136/archdischild-2014-307228 26153506

[26] Grimberg A, DiVall SA, Polychronakos C, Allen DB, Cohen LE, Quintos JB, et al. Drug and Therapeutics Committee and Ethics Committee of the Pediatric Endocrine Society: Guidelines for growth hormone and insulin-like growth factor-I treatment in children and adolescents: growth hormone deficiency, idiopathic short stature, and primary insulin-like growth factor-I deficiency. Horm Res Paediatr. 2016;86:361–397. doi: 10.1159/000452150 27884013

[27] Perez-Colon S, Lazareva O, Purushothaman R, Malik S, Ten S, Bhangoo A. Baseline IGFBP—3 as the Key Element to Predict Growth Response to Growth Hormone and IGF—1 Therapy in Subjects with Non—GH Deficient Short Stature and IGF—1 Deficiency. Int J Endocrinol Metab. 2018;16:e58928. doi: 10.5812/ijem.58928 30197657 PMC6113715

[28] Seino Y, Yamashita S, Morisaki Y, Tanaka H, Chihara K, Tanaka T. Japanese growth prediction model for prepubertal children with growth hormone deficiency. J Pediatr Endocrinol Metab. 2012;25:909–915. doi: 10.1515/jpem-2012-0189 23426820

[29] Spering MA, Majzoub JA, Menon RK, Stratakis CA. Spering Pediatrtic Endocrinology 5^th^ edition. In: Jorge AAI, Grimberg A, Dattami MT, Baron J. Disorders of childhood growth. Bethesda: Elsevier; 2020. pp. 299–256.

[30] David A, Hwa V, Metherell LA, Netchine I, CamachoHübner C, Clark AJ, et al. Evidence for a continuum of genetic, phenotypic, and biochemical abnormalities in children with growth hormone insensitivity. Endocr Rev. 2011;32:472–497. doi: 10.1210/er.2010-0023 21525302

[31] Baron J, Sävendahl L, De Luca F, Dauber A, Phillip M, Wit JM, et al. Short and tall stature: a new paradigm emerges. Nat Rev Endocrinol. 2015;11:735–746. doi: 10.1038/nrendo.2015.165 26437621 PMC5002943

[32] Muzumdar RH, Ma X, Fishman S, Yang X, Atzmon G, Vuguin P, et al. Central and opposing effects of IGF-I and IGF-binding protein-3 on systemic insulin action. Diabetes. 2006;55:2788–2796. doi: 10.2337/db06-0318 17003344

[33] Cianfarani S. Risk of cancer in patients treated with recombinant human growth hormone in childhood. Ann Pediatr Endocrinol Metabol. 2019;24:92–98. doi: 10.6065/apem.2019.24.2.92 PMC6603614 31261472

[34] Bang P, Ahmed SF, Argente J, Backeljauw P, Bettendorf M, Bona G, et al. Identification and management of poor response to growth-promoting therapy in children with short stature. Clin Endocrinol (Oxf). 2012;77(2):169–181. doi: 10.1111/j.1365-2265.2012.04420.x 22540980

[35] Kriström B, Dahlgren J, Niklasson A, Nierop AFM, Albertsson-Wikland K. The first-year growth response to growth hormone treatment predicts the long-term prepubertal growth response in children. BMC Med Inform Decis Mak. 2009; 9:1. doi: 10.1186/1472-6947-9-1 19138407 PMC2651129

[36] Cho WK, Ahn M, Kim EY, Cho KS, Jung MH, Suh B. Predicting first-year growth in response to growth hormone treatment in prepubertal Korean children with idiopathic growth hormone deficiency: Analysis of data from the LG growth study database. J Korean Med Sci. 2020;35(19):e151. doi: 10.3346/jkms.2020.35.e151 32419399 PMC7234860

[37] Yue S, Whalen P, Jee YH. Genetic regulation of linear growth. Ann Pediatr Endocrinol Metabol. 2019;24:2–14. doi: 10.6065/apem.2019.24.1.2 30943674 PMC6449614

[38] Costalonga EF, Antonini SR, Guerra-Junior G, Mendonca BB, Arnhold IJ, Jorge AA. The -202 A allele of insulin-like growth factor binding protein-3 (IGFBP3) promoter polymorphism is associated with higher IGFBP-3 serum levels and better growth response to growth hormone treatment in patients with severe growth hormone deficiency. J Clin Endocrinol Metab. 2009;94:588–595. doi: 10.1210/jc.2008-1608 18984657

[39] Braz AF, Costalonga EF, Montenegro LR, Trarbach EB, Antonini SR, Malaquias AC, et al. The interactive effect of GHR-exon 3 and -202 A/C IGFBP3 polymorphisms on rhGH responsiveness and treatment outcomes in patients with Turner syndrome. J Clin Endocrinol Metabol. 2012;97:E671–E677. doi: 10.1210/jc.2011-2521 22278433

[40] Kang HR, Hwang IT, Yang S. Effect of -202 A/C IGFBP-3 polymorphisms on growth responses in children with idiopathic short stature. Ann Pediatr Endocrinol Metab. 2020;25:31–37. doi: 10.6065/apem.2020.25.1.31 32252214 PMC7136511

[41] Leung KC, Doyle N, Ballesteros M, Waters MJ, Ho KK. Insulin regulation of human hepatic growth hormone receptors: divergent effects on biosynthesis and surface translocation. J Clin Endocrinol Metab. 2000;85:4712–4720. doi: 10.1210/jcem.85.12.7017 11134133

[42] Purandare A, Co Ng L, Godil M, Ahnn SH, Wilson TA. Effect of hypothyroidism and its treatment on the IGF system in infants and children. J Pediatr Endocrinol Metab. 2003;16:35–42. doi: 10.1515/jpem.2003.16.1.35 12585338

[43] Meinhardt UJ, Ho KK. Modulation of growth hormone action by sex steroids. Clin Endocrinol (Oxf). 2006;65:413–422. doi: 10.1111/j.1365-2265.2006.02676.x 16984231

[44] Domene H, Krishnamurthi K, Eshet R, Gilad I, Laron Z, Koch I, et al. Growth hormone (GH) stimulates insulin-like growth factor-I (IGF-I) and IGF-I-binding protein-3, but not GH receptor gene expression in livers of juvenile rats. Endocrinology. 1993;133:675–682. doi: 10.1210/endo.133.2.7688291 7688291

[45] Buckway CK, Guevara-Aguirre J, Pratt KL, Burren CP, Rosenfeld RG. The IGF-I generation test revisited: a marker of GH sensitivity. J Clin Endocrinol Metab. 2001;86:5176–5183. doi: 10.1210/jcem.86.11.8019 11701674

[46] Midyett LK, Rogol AD, Van Meter QL, Frane J, Bright GM, M. S. Study Group. Recombinant insulin-like growth factor (IGF)-I treatment in short children with low IGF-I levels: first-year results from a randomized clinical trial. J Clin Endocrinol Metab. 2010;95:611–619. doi: 10.1210/jc.2009-0570 19880790

[47] Mohanraj L, Kim HS, Li W, Cai Q, Kim KE, Shin HJ, et al. IGFBP-3 inhibits cytokine-induced insulin resistance and early manifestations of athelosclerosis. Plos One. 2013;8:e55084. doi: 10.1371/journal.pone.0055084 23383064 PMC3557269

[48] Ranke MB. Insulin-like growth factor binding-protein-3 (IGFBP-3). Best Pract Res Clin Endocrinol Metab. 2015;29:701–711. doi: 10.1016/j.beem.2015.06.003 26522455

[49] Ballerini MG, Ropelato MG, Domene HM, Pennisi P, Heinrich JJ, Jasper HG. Differential impact of simple childhood obesity on the components of the growth hormone-insulinlike growth factor (IGF)-IGF binding proteins axis. J Pediatr Endocrinol Metab. 2004;17:749–757. doi: 10.1515/jpem.2004.17.5.749 15237710

[50] Schwarz HP, Birkholz-Walerzak D, Szalecki M, Walczak M, Galesanu C, Metreveli D, et al. One-Year Data from a Long-Term Phase IV Study of Recombinant Human Growth Hormone in Short Children Born Small for Gestational Age. Biol Ther. 2014;4:1–13. doi: 10.1007/s13554-014-0014-4 24676989 PMC4254863

